# A Systematic Review of Aphantasia: Concept, Measurement, Neural Basis, and Theory Development

**DOI:** 10.3390/vision8030056

**Published:** 2024-09-22

**Authors:** Feiyang Jin, Shen-Mou Hsu, Yu Li

**Affiliations:** 1 Applied Psychology Program, Department of Life Sciences, BNU-HKBU United International College, Zhuhai 519087, China; sofiajin0830@gmail.com; 2 Department of Applied Social Sciences, The Hong Kong Polytechnic University, Hong Kong, China; 3 Imaging Center for Integrated Body, Mind and Culture Research, National Taiwan University, Taipei 10617, Taiwan; smhsu@ntu.edu.tw; 4 Guangdong Provincial Key Laboratory of Interdisciplinary Research and Application for Data Science, BNU-HKBU United International College, Zhuhai 519087, China

**Keywords:** aphantasia, visual imagery, mental imagery, vividness, cognitive functioning, mental health

## Abstract

People with aphantasia exhibit the inability to voluntarily generate or form mental imagery in their minds. Since the term “aphantasia” was proposed to describe this, it has gained increasing attention from psychiatrists, neuroscientists, and clinicians. Previous studies have mainly focused on the definition, prevalence, and measurement of aphantasia, its impacts on individuals’ cognitive and emotional processing, and theoretical frameworks synthesizing existing findings, which have contributed greatly to our understanding of aphantasia. However, there are still some debates regarding the conclusions derived from existing research and the theories that were constructed from various sources of evidence. Building upon existing endeavors, this systematic review emphasizes that future research is much needed to refine the definition and diagnosis of aphantasia, strengthen empirical investigations at behavioral and neural levels, and, more importantly, develop or update theories. These multiple lines of efforts could lead to a deeper understanding of aphantasia and further guide researchers in future research directions.

## 1. Introduction

In 1880, Galton first systematically elucidated and studied individual differences in visual imagery, designing specific questions to investigate related phenomena [1]. Visual imagery ability refers to the capacity of individuals to create mental images of objects or scenes that are not in front of their eyes [2], such as closing one’s eyes and imagining a brightly colored apple. Visual imagery has been shown to be closely associated with various cognitive functions and everyday activities. The active generation of visual imagery is an important cognitive ability of humans. However, the human capacity for visual imagery can be considered a spectrum, with some individuals exhibiting exceptionally vivid visual imagery, which is referred to as hyperphantasia [3,4], while other individuals can only produce limited or even deficient visual imagery. In 2015, Zeman and colleagues introduced the term “aphantasia” to describe absent or marked reduced mental imagery in individuals [5].

Presently, investigations into aphantasia concentrate on delineating its nature, assessing its frequency, devising measurement methods, examining its influence on cognitive and emotional functions, identifying related disorders, probing its neural underpinnings, and developing conceptual models. In particular, studies examining cognitive functions primarily explore how aphantasia impacts memory, atemporal and future imagination, spatial imagery, mental rotation abilities, and visual search abilities (e.g., [6,7]). Neuroimaging studies indicate a possible link between the lack of visual imagery and the visual cortex (e.g., [2,7,8]).

Aphantasia is still a relatively new topic in psychological research and has not received widespread attention from researchers. This review aims to summarize the existing empirical studies and theoretical viewpoints on aphantasia. First, our discussion will concentrate on the definition, prevalence, heritability, and assessment of aphantasia. Second, we will synthesize the findings regarding aphantasia’s influence on cognitive functions. Third, we will examine its potential associations with related disorders. Fourth, we will summarize existing evidence on the neural basis of aphantasia. Fifth, we will critically appraise the principal theoretical models. Last, we provide future research prospects for aphantasia, aiming to further guide researchers in possible research directions.

## 2. Literature Retrieval and Screening

**Figure 1:**
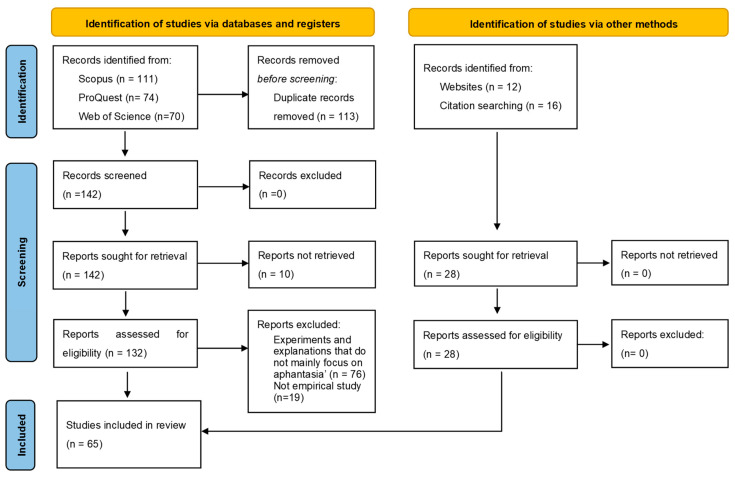
Flow diagram illustrating the research searching and screening.

This study undertook a systematic search of English-language articles published between 2015 and the present (Figure 1). The commencement year of 2015 was selected due to its significance as the year in which Zeman et al. first coined the term “aphantasia” to characterize the absence and marked reduction of visual imagery. The databases used include Scopus, PubMed, ProQuest, and Web of Science. All the relevant journal articles, master’s and doctoral theses, conference papers, and preprints were included in the search. As of 14 August 2024, an initial retrieval yielded 255 literature sources, from which 113 duplicates were removed. Other screening methods and a careful examination of the references cited in pertinent articles added 28 more literature sources to the pool. An in-depth review of titles, abstracts, and full texts for these 170 literature sources led to the exclusion of 10 inaccessible papers, 76 papers not centrally concerned with aphantasia, and 19 non-empirical studies, finally resulting in a curated collection of 65 pertinent literature sources.

We evaluated the quality of these studies and classified their evidence. A study quality evaluation was conducted based on the criteria used in previous works [9,10]. The details of the criteria for the quality assessment are presented in Table S1 (Supplementary Materials). Out of the 65 studies included, 58 studies were cross-sectional or case-control studies. Seven studies were case reports or ecological studies. The overall quality scores (%) were categorized as follows: excellent quality (score ≥81%), good quality (between 61 and 80%), fair quality (between 41 and 60%), poor quality (between 21 and 40%), and very poor quality (≤20%). The evaluation results showed that 58 studies were assessed as “excellent” (N = 22) or “good” (N = 36), 8 studies were assessed as “fair”, and only one study was identified as of “poor” quality (Table S2 in Supplementary Materials).

Based on these 65 papers, this review provides a detailed analysis, evaluation, and summary of the existing empirical research on aphantasia. It should be emphasized that the key studies published before 2015, though not using the term “aphantasia”, have also been considered in discussions if necessary. Reviews and commentaries are further incorporated into this study. In this review, we used “aphantasia” to refer to an absence of mental imagery in any modality for consistency, although the majority of the reviewed studies focused on visual imagery deficits. The detailed content is summarized as follows (see Tables S3–S15 in Supplementary Materials for the details of each study included).

## 3. Definition, Measurement, and Prevalence of Aphantasia

Aphantasia is conceptually defined as the inability to generate mental imagery. However, variations exist in the details of the conceptual definitions [11,12]. Blomkvist reviewed various definitions of aphantasia [4,5,6,7,13,14,15,16,17,18,19] and identified three primary areas of disagreement: whether aphantasics exhibit impairments solely in visual imagery, whether a distinction is made between the absence of voluntary and involuntary imagery, and whether aphantasia primarily concerns the generation of imagery [11]. Regarding the first aspect, aphantasia has also been associated with deficits in non-visual modalities (see Section 4.1). Some studies do not emphasize “visual imagery” when describing its absence, instead using the broader term “mental imagery” to refer to various modalities [7,15,18]. It is recommended that the consistent term “aphantasia” be used and that the modality be specified when necessary, such as “auditory aphantasia” [20]. Concerning voluntariness, some researchers explicitly defined aphantasia as the lack of voluntary visual imagery [6,7,17], while others did not specifically address this aspect [14,16]. Krempel and Monzel call for simply using a broader characterization of aphantasia in the field as there is no solid evidence of preserved involuntary imagery in aphantasics [21]. Regarding the third aspect, increasing evidence suggests that aphantasia is linked to other deficits in cognitive and emotional processes (see Section 4).

In measuring visual imagery deficits, most research utilizes scores from the Vividness of Visual Imagery Questionnaire (VVIQ, [22]) as the primary identification and diagnostic tool [12]. The VVIQ comprises 16 questions and employs a 5-point Likert scale. Participants are asked to rate their visual imagery ability on a scale ranging from “No imagery at all, you only ‘know’ that you are thinking of the object” to “Perfectly clear and as vivid as real seeing”, to differentiate between individuals with visual imagery deficits, those with normal imagery abilities, and those with excessive visual imagery [22]. According to the criteria, a score of 16 denotes a complete absence of visual imagery, with a score of 17–32 indicating vague/dim imagery. In recent years, a new reliable method for identifying visual imagery deficits has emerged, known as the binocular rivalry task [13,23]. Prior instructions to imagine one of the images increase the likelihood of perceiving that image during the subsequent binocular rivalry task. Participants with visual imagery deficits exhibit minimal evidence of image-based binocular rivalry priming [13,23]. The VVIQ-2, an extended version of the VVIQ containing 32 items [24], and a variant of the VVIQ have also been used in the field [4]. Recent research has also found that pupillary responses to light can indicate visual imagery deficits, as affected individuals lack an imagery pupillary light response but still exhibit a perceptual pupillary light response [25]. Additionally, rhythmic visual flickering typically induces illusory percepts, and compared to control groups, individuals with visual imagery deficits are less likely to experience complex and vivid illusions [26,27]. These findings further highlight the physiological differences between individuals with visual imagery deficits and control groups, offering an objective measure for assessing visual imagery intensity.

The vividness of the image can be viewed as a continuous characteristic forming a normal distribution curve with aphantasia occupying the left tail and hyperphantasia occupying the right tail. Before the term “aphantasia” was introduced, the most cited work based on a single question about visual imagery indicated a prevalence rate of 2.1–2.7% for a total absence of visual imagery in the general population [28]. In the field, the VVIQ was widely used for measuring the vividness of visual imagery. Different operational definitions have led to varying prevalence rates. Zeman et al. found that the prevalence rate of aphantasia, where imagery is completely absent, was 0.7% (VVIQ = 16), and that of moderate aphantasia with a VVIQ score of 16–23 was 2.6% [4]. Using a VVIQ score of 16–32 covering “totally absent” to “vague/dim” imagery ratings, Dance et al. found a prevalence rate of 3.9% across two separate samples [29]. When using a score of 16, the rate was only 0.8%. Later, Monzel, Vetterlein, and Reuter conducted meta-analyses and found a prevalence rate of 4.8% across all the included studies and 3.5% in studies using the VVIQ [30]. Recently, Takahashi et al. found in a large sample of Japanese individuals that the rate was 0.07% when using a score of 16 and 3.6% when using a score of 17–32 [31]. A study with a large sample of Brazilian university students reported a prevalence rate of 5.9% based on VVIQ scores of 16–32 [32]. However, another study noted a self-reported prevalence rate of 8.9% in the general adult population, but not all self-reported aphantasics showed low imagery scores on the VVIQ, resulting in only 1.5% [33]. Overall, the prevalence rates of aphantasia are inconsistent due to the use of different criteria across studies. If the strictest criterion is used, the rate would be extremely low. It is important to use consistent standards when discussing the prevalence rates.

For the effects of gender, studies have not found any gender differences in the prevalence of aphantasia [29,34]. Although previous studies found that women tend to score higher on object imagery measures compared to men [35,36], this difference is not statistically significant enough to conclude that men are more likely to be aphantasics. Age has also been considered as a factor influencing aphantasia. However, there is no consensus on how age affects aphantasia [34,37]. Additionally, whether the prevalence of aphantasia varies across different professions remains controversial [4,37,38], despite Galton’s initial suggestion that scientists might have weaker visual imagery abilities as a group [1]. This controversy could be explained by Blajenkova et al.’s opinion that scientists may not lack all types of visual imagery but may be specifically deficient in object imagery [39,40]. Similarly, visual artists tend to excel in spatial imagery rather than object imagery [40].

Aphantasia can be either congenital or acquired [41,42]. It is more common in individuals with a family history of lacking visual imagery, suggesting a genetic basis. However, a recent study found no significant genetic association, indicating the need for further research to explore the genetic evidence of aphantasia [43]. Acquired aphantasia can provide insights into the causes and mechanisms of aphantasia. This form of aphantasia can be triggered by various events, including craniocerebral injuries, emotional disorders, stroke, and postoperative complications [41,42,44]. A case study reported a female who developed aphantasia after contracting COVID-19 [45]. Psychological and psychiatric factors should be carefully considered in the assessment of aphantasia [46,47] as mental illnesses such as depression and anxiety can significantly impact the vividness of visual imagery [46].

## 4. Aphantasia and Cognitive Processing

### 4.1. Visual and Non-Visual Imagery Ability

Visual imagery ability is associated with other forms of imagery, such as olfactory and auditory imagery. In a pioneering study by Zeman et al., 10 out of 21 individuals with visual imagery deficits reported impairments in all forms of imagery, including auditory, olfactory, gustatory, tactile, motor, and bodily imagery, to some extent [5]. A certain number of aphantasics reported a lack of all forms of mental imagery, while some only have deficits in visual imagery [31]. In a large-scale study, 54.2% of aphantasics with visual deficits reported deficits in all types of imagery [4]. Similar findings have been confirmed in other studies, revealing that some aphantasics with visual deficits also reported deficits in other forms of mental imagery (34%, [48]; 26%, [6]). Takahashi and Gyoba reported a person with aphantasia who exhibited a complete deficit in various types of images (e.g., visual, olfactory, pain, tactile, gustatory, and somatic images) in vividness and a substantial deficit in auditory images [49]. Furthermore, individuals with hyperphantasia, who exhibit exceptionally vivid visual imagery, tend to experience more vivid imagery in other types of imagery as well [4]. There may be overlapping mechanisms underlying visual and auditory imagery, as many aphantasia participants report weak or absent auditory imagery [50,51,52] and individuals who lack auditory imagery, which is termed “anauralia”, often show visual imagery deficits [51]. However, some aphantasics self-reported higher auditory imagery than the mean score of the control group [53]. Using cluster analysis, Dawes et al. recently found that aphantasia is heterogeneous and has two subtypes: visual aphantasia, which selectively shows an absence of visual imagery, and multisensory aphantasia, which shows an inability to generate any sensory modality of mental imagery [54].

Overall, some individuals with aphantasia may also experience deficits in all types of mental imagery. However, there is still a lack of large-scale studies to establish a reliable and consistent picture, as well as plausible explanations of the neural mechanisms for understanding these phenomena. It is worth noting that most of the previous measurements of visual and other forms of mental imagery in research have relied on self-report questionnaires (e.g., the VVIQ, the Auditory Imagery Questionnaire, and the Questionnaire upon Mental Imagery). The latest research revealed no significant difference between aphantasics and the control group in an auditory imagery task, even though most of the aphantasic participants reported an impairment in auditory imagery [53]. This suggests that, although self-report questionnaires are convenient for large samples, there may be a meta-cognitive effect in the data. Participants may not actually lack genuine mental imagery but rather report deficits based on cognitive differences and misunderstandings of the questionnaire items. More precise, reliable, and objective measurement methods could help clarify the relationships between different forms of mental imagery deficits by mitigating the risks of participants’ meta-cognitive influences. Alternatively, when using self-report questionnaires, participants should be provided with clear explanations and clarifications of the items to minimize misunderstandings about the content of the questionnaires.

### 4.2. Aphantasia and Memory

Existing research on the impact of visual imagery impairment on memory has mainly focused on working memory, episodic memory, autobiographical memory, and object and spatial memory. Overall, aphantasics do not exhibit deficits in simple working memory tasks [55,56,57,58]. However, it has been reported that aphantasics perform worse than control groups in tasks requiring fine-grained visual working memory, which are cognitively more demanding and require participants to remember smaller differences [55,56,58]. Jacobs et al. reported an aphantasia individual who performed worse than the controls only on the most difficult visual working memory trials requiring a high level of precision [15]. The difficulty level appears to be a crucial factor in visual working memory tasks. In terms of episodic memory, individuals with aphantasia demonstrated significantly lower performance than the control group [6,59,60]. Similarly, aphantasia individuals have been reported to experience difficulties in autobiographical memory [4,5,6,16,55,60,61,62]. Regarding spatial memory, which involves the processing of an object’s location information, two recent studies did not find any deficits in aphantasics [6,17]. For object memory, aphantasics showed poorer performance in object memory in a drawing task requiring object processing [17]. Siena and Simons used both subjective and objective measures of object and spatial memory and found that aphantasia participants showed no objective deficits in memory performance, indicating that some aphantasics might show a deficit in the awareness of mental imagery [63].

It should be noted that new measurements of memory performance have been used in existing research, deviating from traditional scales and experimental tasks. For instance, drawing has been used to complement the assessment of memory performance in participants [17]. In the case of drawing, aphantasics and control groups were instructed to draw scene images from real-world memory. It was found that aphantasics remembered fewer objects and used fewer colors to draw, and relied more on verbal scaffolding to compensate for the lack of visual imagery [17].

Overall, memory is a relatively well-researched aspect in the field of aphantasia. Many studies have employed questionnaires and experiments to measure participants’ memory performance. In visual working memory, aphantasics exhibit similar performance to control groups, which could be explained by their potential use of semantic encoding or other representational strategies to aid them in completing working memory tasks [7]. However, some studies indicate that aphantasics perform worse in these visual working memory tasks [15]. The inconsistency of these findings may be attributed to task characteristics, measurement methods, and individual differences among participants. For instance, the heterogeneous outcome included only one individual with aphantasia, suggesting that the observed differences in working memory could be influenced by sample size or the unique characteristics of the case study [15]. As for autobiographical memory, all the results seem to converge on the conclusion that individuals with aphantasia have poorer autobiographical memory. Differences in object memory and spatial memory performance between the aphantasics group and the control group may be related to their abilities in object imagery and spatial imagery. These differences may be explained through the neural mechanisms of the ventral and dorsal pathways of visual processing, which will be discussed more comprehensively later.

### 4.3. Aphantasia and Object and Spatial Imagery

Object imagery refers to visualizing the image appearance of objects and scenes, such as their shape, color, and brightness, while spatial imagery refers to visualizing the spatial relationships and movements of objects and their components, and spatial transformation [64]. Existing research on the performance of individuals with aphantasia in object and spatial imagery abilities has not reached a consensus and can be categorized into three types of studies. The first type of study indicates that aphantasia individuals score lower in object imagery abilities but show no significant differences from control groups in spatial imagery performance [6,17,52,60]. The second type of study shows that aphantasics score significantly lower in object imagery than control groups but perform better in spatial imagery tests [13,55]. In the last category of results, individuals with aphantasia demonstrate poorer performance in both object and spatial imagery abilities [65,66]. Based on these findings and a review of the literature, researchers have proposed two types of visual imagery impairments: object aphantasia and spatial aphantasia [39,67].

The finding that object imagery is impaired while spatial imagery remains unaffected can be also explained by the functional division of visual pathways. Aphantasics’ ventral pathway for object processing may be impaired, while the dorsal pathway for spatial processing remains intact ([7,68], also see below). To better explain spatial aphantasia, researchers have proposed that this type of aphantasia may be related to functional changes in the visual dorsal stream projecting to the frontal lobe [67]. However, this explanation still lacks further support from neuroimaging studies.

### 4.4. Aphantasia and Atemporal and Future Imagination

Atemporal and future imagination are related to voluntarily imagining general events and future events, respectively. Autobiographical interviews on atemporal and future imagination show that aphantasia participants have poorer abilities in atemporal and future imagination, with fewer details in their imagined scenarios [16,60]. Evidence suggests that atemporal and future imagination abilities are related to autobiographical memory [16]. This may help explain why aphantasics show poorer abilities in atemporal and future imagination, as their autobiographical memory abilities are also lower compared to control groups.

### 4.5. Aphantasia and Mental Rotation Task Performance

The mental rotation task has been widely used to investigate individuals’ spatial abilities [69]. It has been reported that, compared to control groups, individuals with aphantasia showed equal [16,53,70] or even higher accuracy in mental rotation tasks [55,65,66,71]. However, aphantasics also required longer response times in these tasks [58,65,71].

Aphantasia and control groups performed similarly in mental rotation tasks, possibly due to the use of different strategies to solve the tasks [66,71]. Pounder and his colleagues suggested that aphantasics may employ non-visual processes in these tasks [58]. For example, the patient in Furman et al.’s study self-reported using a spatial and kinesthetic strategy instead of low-level visual object imagery in the mental rotation task [13,66]. Specifically, he used a first-person path-following strategy (i.e., spatial strategy) containing kinesthetic features, using his body as a reference. This hypothesis is supported by the performance of congenitally or early blind individuals in mental rotation tasks, as they did not show differences from control groups in completing complex spatial tasks, indicating that mental rotation tasks can be completed independently of visual processes [72]. Kay and his colleagues suggested that aphantasics are more likely to use analytic strategies, which do not depend on features and orientation [71]. Additionally, the mental rotation task is considered to rely on an individual’s spatial imagery ability, and many individuals with aphantasia report having similar or even better spatial imagery abilities compared to control groups [13,60].

### 4.6. Aphantasia and Visual Searching Ability

Visual search is a commonly used skill in everyday life that helps us find the things we need, even if the desired target is within our current field of vision. When performing visual search tasks, aphantasics exhibit significantly slower speeds compared to control groups [73,74]. This difference could be explained by the varying involvement of visual imagery in top-down strategies between the two groups [74,75]. However, in the Moriya’s task, which examines participants’ attentional guidance in visual search tasks ([76]; participants are first asked to imagine a color primed by a color word such as “blue” and then to indicate whether one of the two colored squares presented on the left and right sides has an opening at the top or bottom), the priming process seems to be similar between the aphantasia group and the control group [73]. This can be explained by the characteristics of the task, where the instructions in the Moriya’s task are overly complex, resulting in a lack of visual priming and only non-visual priming [73]. These findings suggest that visual imagery could affect the way people perceive the world. Future research would benefit from designing materials that are more ecologically valid, such as incorporating environmental cues into the experimental design.

## 5. Aphantasia and Disorders and Emotional Processing

### 5.1. Emotion

Mood may influence the vividness of mental imagery [4]. By contrast, mental imagery was also found to affect individuals’ emotion experience. Due to absent or reduced visual imagery, aphantasics experience reduced emotional engagement and less sympathy for characters in stories [77] and exhibit lower levels of fear response when reading scary materials [78]. Mental imagery ability was found to be positively associated with empathy elicited by descriptions, but not associated with empathy elicited by pictures [79]. Additionally, aphantasics seem to experience lower emotional intensity when listening to music [80]. This may be because vivid imagery makes thoughts more realistic, triggering stronger responses in the brain’s emotional circuitry and thereby amplifying emotions [81]. Future research can continue to explore different types of emotions and incorporate neuroimaging methods to validate previous findings.

### 5.2. Mental Health

Although aphantasia may impact individuals’ cognition and emotional processing and potentially cause some personal distress, it is not currently included in any common clinical diagnostic systems. Furthermore, the impact of aphantasia on individuals’ daily lives and personal distress is relatively minimal and not sufficient to classify it as a psychological disorder [30,82]. However, research has found associations between aphantasia and certain conditions or diseases [83]. Moreover, scientists have proposed that progressive aphantasia may be a precursor to dementia [84].

Existing research has focused on the impact of aphantasia on mental health, such as depression, anxiety, distress, and well-being. Research has revealed that the aphantasia group showed no significant differences compared to the control group in terms of depression, anxiety, and state-trait anxiety [16,81]. However, Monzel, Vetterlein, and Reuter found that 34.7% of aphantasia participants reported distress caused by the lack of visual imagery [30]. Nevertheless, the researchers also suggested that these negative emotions were relatively weak and did not significantly affect individuals’ daily lives [30].

### 5.3. Post-Traumatic Stress Disorder (PTSD)

Previous research has established a link between mental imagery and PTSD, a condition characterized by re-experiencing traumatic events through unwanted and recurring intrusive memories and nightmares [85,86]. Aphantasics have been found to experience fewer intrusions and exhibit less avoidance behaviors following trauma [6], both of which are predictors of PTSD. Additionally, the aforementioned lack of visual imagery and its association with reduced emotional intensity may also contribute to their lower likelihood of developing PTSD. However, it is important to note that researchers emphasize the limited protective effect of visual imagery deficits when individuals with such deficits face stressful events in their daily lives [6].

### 5.4. Autism

Individuals with aphantasia frequently score higher on the Autism Quotient Questionnaire (AQ) and are more likely to be classified within the autism spectrum [16,87]. This association may be related to the connection between imagery and mentalizing. Compared to control groups, aphantasics exhibit slightly reduced atemporal and future imagination, as well as episodic memory [6,16,60], which are also characteristics of autistic spectrum disorder [88,89]. Additionally, limited or absent visual imagery can impair the theory of mind ability in aphantasics, leading to difficulties in social skills [87]. These factors may explain why individuals with aphantasia tend to have higher scores on the AQ, indicating a potential connection between visual imagery deficits and autistic spectrum disorder.

### 5.5. Prosopagnosia

Aphantasia has been associated with reduced facial recognition ability [31]. In a recent study, 5.9% of participants in the spatial aphantasia group were identified as having prosopagnosia [67], and face recognition difficulties were found to be more common in individuals with aphantasia [16,90]. Aphantasics also reported difficulties with facial recognition tasks [41]. Additionally, aphantasics have been associated with lower confidence in tasks involving facial perception [91]. In a study of developmental prosopagnosia, about 20% of participants with developmental prosopagnosia reported a comorbidity with aphantasia [92]. However, aphantasia does not significantly impact accuracy in tasks involving the construction of facial composites and face recognition tasks [93], as individuals with aphantasia can still create facial composites from memory similarly to control groups [90]. Nevertheless, some researchers have argued that the scores of aphantasics in tests are not sufficient to diagnose them with prosopagnosia [66]. Overall, individuals with aphantasia tend to exhibit poorer face recognition abilities. However, this may be influenced by the scales used in experiments, as these scales may elicit meta-cognitive effects [16]. In other words, individuals with aphantasia may have reduced confidence in their own facial recognition abilities due to their lack of vivid facial imagery, which can manifest as relatively lower scores on self-report measures.

## 6. The Neural Basis of Aphantasia

Although numerous studies have focused on the neural basis of mental imagery [7,8,94,95], there are limited studies utilizing modern neuroimaging techniques such as magnetic resonance imaging (MRI) and electroencephalography (EEG) to explore the neural basis of visual imagery deficits. Using resting-state and task-based MRI, Milton et al. recently compared the brain activities of three groups: aphantasics, typical controls, and hyperphantasics [16]. The results showed that individuals with hyperphantasia exhibit stronger connectivity between the prefrontal regions and visual-occipital network compared to the aphantasia group. When comparing the visualization and perception of famous faces and places, individuals with hyperphantasia and the control group exhibited greater frontal and parietal activation compared to the aphantasia group [16]. Another functional MRI study showed that the aphantasic group exhibited decreased activation in the hippocampus and increased activation in the visual-perceptual cortex during an autobiographical memory task [96]. However, the control group displayed strong negative task-based functional connectivity between the hippocampus and the visual cortex during the task, and the resting-state functional connectivity between these two areas could predict visualization skills [96]. Furthermore, using transcranial magnetic stimulation to induce changes in brain activity, Dupont, Papaxanthis, Madden-Lombardi and Lebonet found that there was no increase in the amplitude of motor-evoked potentials triggered in the target right index finger in the aphantasia group, indicating a lack of corticospinal excitability in individuals with aphantasia during motor simulation [97]. Similar findings were also observed in an action reading task, which involved motor simulation [98].

Case studies are also a crucial source of neural evidence. Lesion studies have found that patients showed intact visual imagery after brain lesions restricted to the occipital cortex [99,100,101,102], suggesting that early visual areas are not involved in visual imagery. Moreover, patients with damage to the anterior part of the temporal lobe, particularly in the left hemisphere, often report an inability to generate visual images [103,104,105], which is consistent with observations of a left hemisphere bias [106]. Zeman et al. reported a patient who showed reduced activation in the occipitotemporal regions during an imagery task [107]. In a recent study, a patient with an absence of visual imagery ability showed selective lesions in a specific area of the left fusiform gyrus and a portion of the right lingual gyrus, demonstrating a causal role of the left fusiform gyrus in visual imagery [41]. Therefore, the fusiform region “might act as a neural interface between sensory information coming from the occipital cortex and semantic processing in the anterior temporal lobe” during visual perception, and “could endow semantic memories with visual information during visual imagery” ([100], p. 517). An EEG study with source reconstruction reported that during a visual imagery task, an aphantasic begins the evoking phase from the left temporal area while lacking activation of the occipital and parietal lobes, which are associated with visual image vividness [66].

Due to the limited evidence in these studies, it is challenging to draw a consistent picture of the aberrant brain activity in aphantasia. However, we can still draw inspiration from neuroimaging studies based on individual differences in mental imagery. Spagna et al. recently conducted a meta-analysis of fMRI studies of visual imagery and found that visual imagery recruits several fronto-parietal areas and a specialized area in the left fusiform gyrus [94]. The specialized area was labeled the fusiform imagery node (FIN), referring to a brain network node specifically responsible for voluntary visual mental imagery [94,108,109]. Liu et al. found that imagery tasks activated the left frontal–parietal regions, the FIN, and areas in the ventral temporal cortex, which were similarly activated in the aphantasia and control groups [110]. However, the connectivity between the FIN and the frontoparietal regions is reduced in aphantasics [110]. Together, these brain-lesion cases and fMRI studies demonstrate that the fusiform gyrus is a core area for visual imagery, and damage to or impairment of it could lead to deficits in visual imagery. Additionally, abnormalities in the fusiform gyrus have been associated with prosopagnosia, which may explain the facial recognition deficits observed in individuals with visual imagery disorders [111,112].

A related debate concerns the involvement of the early visual cortex in (impaired) visual imagery [100,113]. Using stimulation, Kosslyn et al. found that the early visual cortex is causally involved in visual imagery [114]. Bergmann et al. documented that a smaller V1 size is associated with stronger but less precise imagery, indicating an anatomical basis [115]. Keogh et al. suggested a causal relationship between cortical excitability in the early visual cortex and the intensity of visual imagery [116]. However, Meng et al. suggested that an imagery-related representation exists in the primary visual cortex of aphantasics, despite the absence of visual imagery, though the representation contains less or transformed sensory information [117]. The results from Cabbai et al. showed a dissociation between V1 representations and subjective imagery [118]. Bartolomeo et al. [100] argued that the left fusiform gyrus plays a crucial role in visual imagery, rather than the early visual cortex, and that the involvement of the early visual cortex [114] might be modulated by downstream areas. Dijkstra recently reviewed existing evidence and proposed that “imagery can recruit the early visual cortex, but that does not mean that it always does so” [95].

## 7. Theory Development

Individuals with imagery deficits can still have imagination capabilities similar to those without such deficits, such as creating novels and movies. Based on this, Arcangeli attempts to distinguish between mental imagery and sensory imagination [119]. Mental imagery can be considered a type of mental content (e.g., the appearance of an apple). In contrast, sensory imagination is a special psychological attitude that involves the recreation of perceptual experiences. According to this theory, most individuals previously defined as having aphantasia in past research may actually have a deficiency in sensory imagination rather than in mental imagery, which could explain why individuals with visual imagery deficits can still perform imaginative tasks.

Nanay explains aphantasia from the perspective of both conscious and unconscious visual imagery [18]. Previous research has found that individuals with aphantasia do not exhibit the imagery priming effect in binocular rivalry tasks; yet some participants can generate vivid dreams and perform visual imagery tasks similarly to control groups. To explain this phenomenon, Nanay distinguishes between conscious and unconscious visual imagery abilities, both of which could be generated voluntarily or involuntarily [18]. One type of aphantasia involves a fundamental lack of visual imagery, while the other type involves the ability to generate visual imagery without conscious awareness [73]. However, this theory is challenged by Blomkvist [11], who argues that it does not fully explain the issues of episodic memory or imagination of atemporal and future events in visual imagery deficits, as it does not provide a link between mental imagery and the episodic processes in episodic memory and episodic imagination. Additionally, there is research suggesting that imagery tasks can be accomplished through cognitive strategies that do not rely on imagery [91,120,121,122]. A recent study using both implicit and explicit priming tasks found that participants with aphantasia did not show priming effects, suggesting that aphantasia is an inability to generate visual imageries rather than an impairment in conscious awareness of images [123].

To provide a better explanation for aphantasia, Blomkvist proposed enhancements to the Constructive Episodic Simulation Hypothesis (CESH) model [11]. The original CESH posits that memory and imagination involve three key processes: the semantic retrieval process, episodic retrieval process, and (re)combination process [124]. Blomkvist expanded on this model by adding three new components: “memory indices, differing episodic retrieval mechanisms for all kinds of sensory information, and spatial retrieval mechanisms” [11]. These additions aim to provide a new theoretical explanation for visual imagery deficits. According to this updated theory, the mechanism of the episodic system is deficient, resulting in the loss of visual imagery. However, this theory still lacks empirical research data to support it further.

The theoretical framework of the ventral and dorsal pathways in the visual system has been proposed for many years [125,126,127] (although there are ongoing debates about this framework, [128,129]. Pearson proposed that the ventral (or “what”) pathway is associated with object information, while the dorsal (or “where”) pathway is associated with location and spatial features [7]. The two aspects of visual imagery, object imagery and spatial imagery, are also likely generated through the ventral and dorsal pathways [39]. Damage to the ventral pathway could impair individuals’ ability to visualize the appearance of objects, while damage to the dorsal pathway is associated with a disrupted ability in spatial imagery. These processes can be dissociated in aphantasia, which may explain why individuals with visual imagery deficits perform similarly to, or even better than, control groups in spatial imagery tasks [7,68]. Bergmann and Ortiz-Tudela also suggested that visual feedback pathways used during episodic and schematic memory retrieval may be different depending on the two visual processing streams: episodic memory retrieval involves both the “what” and “where” streams, while schematic memory retrieval primarily involves the “where” stream [130]. In other words, aphantasia may be associated with differences in the “what” stream.

Inspired by neural models of mental imagery [7,8] and existing sources of neural evidence, Zeman tried to depict candidate neural mechanisms of extreme imagery, aphantasia, and hyperphantasia [2]. In the brain mapping picture, there are five functional clusters, each involved in a unique role in mental imagery: the frontal cortex for initiating imagery generation, the parietal cortex for interacting with the frontal cortex to generate imagery during which attentional and spatial aspects of imagery are mediated, the temporal cortex, including limbic structures for enabling access to the semantic and episodic memories that determine what to visualize, and higher-order visual areas (e.g., the FIN) for visualizing imagery. The extent to which activity in the early visual cortex (e.g., the V1) is required for imagery is still debated. Moreover, the connections between these clusters are also essential for imagery generation. From this mapping picture, Zeman [2] proposes five candidates showing neural atypicality in extreme imageries: variations in the strengths of the top-down feedback connection between higher-order regions (the frontal cortex) serving as cognitive control and modality-specific areas (e.g., the visual cortex) activated by sensory imagery; variations in the structure and function of the frontal cortex involved in generating imagery; variations at the level of higher-order visual areas, including the FIN proposed by Spagna et al. [94]; variations in the anatomical structure of the early visual cortex, including the V1; and variations in the saliency involving the parietal areas of the frontoparietal control system. In this proposal, Zeman provided future directions for researchers in the field [2]. A neural model constructed and refined from accumulated evidence would add to a better understanding of aphantasia.

## 8. Summary and Future Directions

This review primarily examines the definition, prevalence, and measurement methods of aphantasia, as well as its impacts on individual cognitive and emotional processing, associated disorders, neural basis, and theory development. Research on aphantasia is continuously progressing, and its descriptions are becoming increasingly clear. However, several debated areas remain, and the study of its impacts on individuals and underlying mechanisms faces several challenges. Future investigations into aphantasia should focus on the following aspects.

### 8.1. Clarify Definition and Diagnosis

It is evident that the current research on aphantasia is still in its early infancy. One of the primary concerns is the definition of aphantasia. Clear definitions serve as the foundation for advancing further research and facilitating effective communication among researchers. The existing literature presents variations in the definition of aphantasia [12]. For example, some definitions do not explicitly distinguish between voluntary and involuntary imagery (see a review by [21]). The cut scores for the diagnosis of aphantasia vary across studies (see Section 3). The inconsistent use of the arbitrary cut scores is considered a hindrance to cross-study comparison and communication among researchers. Moreover, the use of cut scores may also discourage efforts to examine individual differences in imagery. Additionally, more effective, objective tools for non-clinical and clinical purposes should be developed [131,132] rather than relying predominantly on the VVIQ or its variants [133].

The terminology in the field has recently been debated [20,134,135,136]. While the debate continues on whether the term “aphantasia” specifically refers to the absence of visual mental imagery or encompasses a deficit of all types of mental imagery (see Section 3), most current research on aphantasia focuses on visual imagery. Other terms have been proposed to refer to an absence of other modalities of imagery. For auditory imagery, Hinwar and Lambert introduced the term “anauralia” to refer to an absence of auditory imagery and “hyperauralia” to refer to the experience of extremely vivid auditory imagery [51]. Dance, Ward, and Simner proposed the term “dysikonesia” to refer to multisensory or global aphantasia [48]. In this context, Monzel, Mitchell et al. believe that these new terms complicate the field, making communication less effective for researchers and the public, and therefore advocate for the use of the simple term “aphantasia,” which is widely known [20]. When referring to an absence of a specific modality of imagery, it is easy to use modality-specific terms (e.g., “visual/auditory/multisensory aphantasia”).

These divergencies across studies create difficulties in the classification of participants and scientific communications among researchers and the public, therefore hindering progress toward a better understanding of aphantasia. Researchers in the field should reach a consensus on these issues to facilitate further development.

### 8.2. Strengthen Behavioral Research

Aphantasia research is still at its early stages, and there is limited literature in several research directions. Future studies should build upon existing efforts to systematically examine this phenomenon to better understand aphantasia. At the behavioral level, more research is urgently needed within and beyond existing research directions. First of all, together with a clear and consistent definition of aphantasia, researchers should focus on examining the nature of aphantasia, including but not limited to investigating effects of demographic variables (e.g., age and gender) and their interactions in large datasets, developing new assessment or diagnostic tools with high reliability and validity, elucidating perceptual and cognitive processes required by tasks used to elicit mental imagery, and determining the comorbidity rate of two or more subtypes of aphantasia in large samples. Second, researchers should investigate how aphantasia is associated with other psychological aspects (e.g., conscious experience, [18,137,138,139], the influences of aphantasia on emotional processing and disorders (see Section 4), and possible mechanisms underlying these relationships (e.g., [140]). Third, research on hyperphantasia, the opposite extreme characterized by vivid mental imagery, can also help contribute to a better understanding of aphantasia. Viewing mental imagery ability as a continuum could be more realistic and scientifically useful in understanding the absence of or reduction in imagery in people with aphantasia. Fourth, researchers should use these different sources of evidence as a foundation to propose or create intervention programs for people with aphantasia. The effectiveness of these interventions can also help researchers better understand the nature of this phenomenon, fostering further advances.

### 8.3. Discover Neural Bases

Unraveling the neural bases of aphantasia has recently become a prominent research topic. Neuroimaging evidence has been instrumental in understanding the manifestations of aphantasia and in constructing neural models. Employing neuroimaging methods, future research can explore various variables to gather more neural evidence. Specifically, researchers can utilize the high spatial resolution of MRI techniques to investigate regional activation and inter-regional connections (structural and functional connectivity) involved in the absence or reduction of mental imagery. Previous functional MRI studies have identified areas involved in mental imagery, based on which neural models have also been proposed (e.g., [14,115,116]; see a review by [7]). Although some studies of aphantasia have been conducted [16,107,110], it is difficult to accumulate convergent evidence for localizing aberrant brain regions and connections. Using EEG and magnetoencephalography (MEG) techniques with high temporal resolution [141,142], researchers can examine the neural dynamics of the processes involving mental imagery in people with and without aphantasia (e.g., [66]). For example, Xie et al. found shared alpha-band neural representations in visual imagery and perception [143]. Combining spatial and temporal neural evidence across different studies, or even using fusion techniques (e.g., EEG-MRI fusion, [144]) can contribute to a better understanding of aphantasia by incorporating different types of information into a single picture, further facilitating the construction of neural models. Recent technical advances in multi-variate pattern analysis (e.g., [145,146]) may also be powerful in understanding the neural correlates of aphantasia, as demonstrated in previous studies of mental imagery [111,117,143]. Non-invasive brain stimulation techniques [147] can be powerful tools for discovering causal evidence of the involvement of brain areas in mental imagery. Together, the convergence of behavioral and neural evidence not only helps elucidate various aspects of aphantasia but is also useful in building behavioral and neural models that explain this phenomenon [2]. In addition, evidence from other sources of investigation should also be encouraged. It has been reported that aphantasia has a genetic basis [4] and that dopamine plays an important role in generating mental imagery [148]. 

### 8.4. Construct and Refine Theories

Good theories help humans systematically and scientifically understand their minds [149,150]. Although aphantasia has garnered increasing attention over the past decade, and has also gained interest in art, philosophical, and theoretical discussions [80,83,151,152,153,154,155,156,157,158,159,160], empirical studies examining this phenomenon remain limited. The field is still in its early stages. A lack of theories for synthesizing the existing literature is one of the field’s significant characteristics. Although some accounts and models have been proposed to interpret behavioral findings [11,18,119], they have focused on certain aspects. Neural models for synthesizing neural evidence (e.g., [2]) are largely unknown or require further examination. One of the main reasons is that both behavioral and neural empirical studies are very limited in number and diversity (see above), resulting in insufficient evidence to be integrated into a theory or model.

In addition to proposing new theories or models, we advocate for efforts to use existing theories or models of mental imagery [7,8,11,109] as a foundation to theoretically guide future directions on researching aphantasia. Modifications to these models or theories will be made as more evidence is collected. We believe these changes will either refine existing theories or help foster new ones. For instance, theories that explain the mechanisms of visual imagery can be adapted to account for the absence of such imagery in individuals with aphantasia. By modifying these models to include the cognitive and neural underpinnings of aphantasia, researchers can create a more robust theoretical framework that encompasses both the presence and absence of mental imagery.

Given that aphantasia may extend beyond visual imagery to include other modalities, such as auditory or tactile imagery, theories should be developed to address these broader aspects. This could involve creating a unified model that categorizes different types of aphantasia (e.g., visual, auditory, multisensory) and explores how these modalities interact within cognitive processes. Such a model would not only clarify the nature of aphantasia but also facilitate comparisons with individuals who possess typical imagery capabilities.

### 8.5. Encourage Direct and Conceptual Replications

In recent years, concerns about replication in psychology have increased (e.g., [161,162]). Replicability is a crucial indicator of the reliability and stability of research findings [163]. Conducting replicable experiments to demonstrate the replicability of the findings lays a solid foundation for further investigations. There are two types of replications, each with different requirements. Conceptual replication deliberately modifies key elements of the original program to test the robustness of the phenomenon or the generalizability of the theoretical claims, while direct replication involves recreating the original experiment, which has been questioned regarding its impossibility of implementation [164].

The inconsistencies in research findings on aphantasia, which can undermine confidence in the literature, call for the consideration of replicability in existing studies, requiring numerous replication studies to confirm the reliability and stability of results. For example, there are limited studies on the relationship between aphantasia and atemporal and future imagination, as well as visual search. Although consistent conclusions have been reached, different experimental designs are still needed to validate the accuracy and replicability of the results (conceptual replications). Moreover, researchers have explored the relationships between attentional templates [19], sensory sensitivity [48], verbal overshadowing [120], mid-level characteristics of drawings [165], and aphantasia, but these are isolated studies lacking cross-validation from other research, indicating the necessity of conducting direct and conceptual replication studies.

To facilitate replication efforts, aphantasia researchers should adopt open science practices, such as sharing data, materials, and protocols. By making these more transparent, the scientific community of aphantasia can more easily conduct replications and build upon existing work. Open access to the datasets of research published or to be published can also encourage more collaboration and foster a collective effort to understand this phenomenon more deeply.

## 9. Conclusions

In this study, we have provided a comprehensive review of aphantasia, encompassing its definition, prevalence, measurement methods, empirical research, and relevant theories. The existing literature has contributed to a good understanding of aphantasia. The absence of visual imagery can have significant implications for an individual’s cognitive and emotional processing and is also associated with certain psychological disorders. Studies on the neural bases of aphantasia are gradually increasing, especially in recent years. Following these endeavors, theories and neural models have been proposed to summarize existing behavioral and neural evidence. The research of visual imagery deficits is continually growing, although there are still areas of debate. However, a consensus regarding the theoretical framework has not yet been achieved. The theories still lack sufficient empirical support, leaving a significant gap. We have emphasized several points to which researchers can make contributions, such as clarifying diagnoses, strengthening behavioral and neuroimaging research, constructing and refining theories, and encouraging replications. New work on the different aspects of aphantasia is not only beneficial for expanding researchers’ understanding of cognitive and emotional processes and their relationships with other disorders, but is also helpful for building theories or model.

## Data Availability

Not applicable.

## Supplementary Materials

The following supporting information can be downloaded at: https://www.mdpi.com/article/10.3390/vision8030056/s1, Table S1. Criteria for quality assessment of the studies; Table S2. Scores and quality categories of the 65 studies evaluated; Table S3. Prevalence; Table S4. Performance on computer tasks; Table S5. Acquired aphantasia; Table S6. Non-visual imagery ability; Table S7. Memory; Table S8. Object and spatial imagery; Table S9. Atemporal and future imagination; Table S10. Mental rotation task performance; Table S11. Visual search ability; Table S12. Emotion; Table S13. Aphantasia and related disorders; Table S14. Neural Basis of Aphantasia; Table S15. Others.
