# Supplementary material for: A Systematic Review of Aphantasia: Concept, Measurement, Neural Basis, and Theory Development
Source: Vision (Basel). 2024 Sep 22;8(3):56. doi: 10.3390/vision8030056 (PMC11437436; doi:10.3390/vision8030056)
Supplement: Supplementary file 1 [file vision-08-00056-s001.zip › vision-3127036-supplementary.pdf]

vision

Supplementary file

A Systematic Review of Aphantasia: Concept, Measurement, Neural Basis, and Theory Development

Feiyang Jin, Shen-Mou Hsu and Yu Li

Tables

Table S1. Criteria for quality assessment of the studies.

Study design	0, ecological study or case report; 1, cross-sectional or case-control study; 2, intervention or longitudinal study
Aphantasia assessment	0, self-reported or non-validated questionnaires; 1, self-reported validated questionnaires; 2, interviews conducted by experts, physical measurements, or experiments
Outcome assessment	0, self-reported or non-validated questionnaires; 1, self-reported validated questionnaires; 2, interviews conducted by experts, physical measurements, or experiments
Variable multiplicity	0, exposure of interest one of the many variables tested; 1, exposure of interest the main variable tested
Confounding factors	0, no confounding variables evaluated; 1, confounding variables evaluated, but several major confounders ignored; 2, confounding variables carefully considered and evaluated
Potential bias	0, other research design or conduct flaws that might have contributed to bias; 1, no other serious study issues
Statistical analysis	0, errors in or incorrect statistical testing or interpretation of statistical tests, which may have influenced results; 1, appropriate statistical analysis and interpretation of results
Effect size	0, incomplete information; 1, complete information (e.g., estimate and confidence interval or standard error)

Table S2. Scores and quality categories of the 65 studies evaluated.

	Study Design (0–2)	Aphantasia Assessment (0–2)	Outcome Assessment (0–2)	Variable Multiplicity (0–1)	Confounding Factors (0–2)	Potential Bias (0–1)	Statistical Analysis (0–1)	Effect Size (0–1)	Score (Absolute)	Score (%)	Quality Category
Bainbridge et al., 2021 [17]	1	1	2	1	2	1	1	1	10	83	Excellent
Balas, 2024 [165]	1	0	2	1	0	1	1	0	6	50	Fair
Beran et al., 2023 [33]	1	1	2	1	1	1	1	1	9	75	Good
Bumgardner et al., 2021 [44]	0	1	NA	1	1	1	NA	NA	4	50	Fair
Cabbai et al., 2024 [118]	1	1	2	1	1	1	1	NA	8	73	Good
Crowder, 2018 [65]	1	1	2	1	1	1	1	1	9	75	Good
Dance et al., 2023 [90]	1	1	2	1	2	1	1	1	10	83	Excellent
Dance et al., 2022 [29]	0	1	NA	1	2	1	1	1	7	70	Good
Dance et al., 2021 [48]	1	1	1	1	2	1	1	1	9	75	Good
Dance et al., 2021 [87]	1	1	1	1	2	1	1	1	9	75	Good
Dando et al., 2023 [59]	1	1	2	1	2	1	1	1	10	83	Excellent
Dawes et al., 2020 [6]	1	1	1	1	2	1	1	1	9	75	Good
Dawes et al., 2024 [54]	1	1	1	1	1	1	1	0	7	58	Fair
Dawes et al., 2022 [60]	1	1	2	1	2	1	1	1	10	83	Excellent
Dupont et al., 2024 [97]	1	1	2	1	2	1	1	1	10	83	Excellent

Dupont et al., 2024 [98]	1	1	2	1	2	1	1	1	10	83	Excel-lent
Fielding et al., 2020 [38]	1	1	1	1	1	1	1	1	8	67	Good
Fulford et al., 2018 [14]	1	1	2	1	2	1	1	NA	9	82	Excel-lent
Furman et al., 2022 [66]	0	1	2	1	2	1	1	NA	8	73	Good
Gaber et al., 2021 [45]	0	0	NA	1	1	1	NA	NA	3	38	Poor
Ganczarek et al., 2020 [55]	1	1	2	1	0	1	0	0	6	50	Fair
Gouveia, 2023 [32]	1	1	0	1	1	1	0	0	5	42	Fair
Gulyas et al., 2022 [34]	0	1	NA	1	2	1	1	1	7	70	Good
Hashim et al., 2024 [80]	1	1	1	1	1	1	1	1	8	67	Good
Hinwar & Lambert, 2021 [51]	1	1	1	1	1	1	1	1	8	67	Good
Jacobs et al., 2018 [15]	1	1	2	1	2	1	1	1	10	83	Excel-lent
Kay et al., 2024 [71]	1	1	2	1	1	1	1	1	9	75	Good
Kay et al., 2022 [25]	1	2	2	1	1	1	1	NA	9	82	Excel-lent
Keogh & Pearson, 2024 [23]	1	2	NA	1	1	1	1	0	7	70	Good
Keogh & Pearson, 2021 [19]	1	2	2	1	2	1	1	0	10	83	Excel-lent
Keogh & Pearson, 2018 [13]	1	1	2	1	1	1	1	0	8	67	Good
Keogh et al., 2023 [81]	1	1	1	1	1	1	1	0	7	58	Fair
Keogh et al., 2021 [56]	1	2	2	1	2	1	1	1	11	92	Excel-lent

Knight & Milton, 2022 [57]	1	1	2	1	1	1	1	1	9	75	Good
Knowles et al., 2021 [42]	0	1	NA	1	1	1	1	0	5	50	Fair
Konigsmark et al., 2021 [26]	1	1	2	1	2	1	1	1	10	83	Excellent
Liu & Bartolomeo, 2023 [91]	1	1	2	1	1	1	1	1	9	75	Good
Liu et al., 2023 [110]	1	2	2	1	2	1	1	NA	10	91	Excellent
Meng et al., 2023 [117]	1	1	2	1	1	1	1	NA	8	73	Good
Milton et al., 2021 [16]	1	1	2	1	2	0	1	NA	8	73	Good
Monzel et al., 2024 [120]	1	1	1	1	2	1	1	1	9	75	Good
Monzel & Reuter, 2024 [74]	1	1	2	1	2	1	1	1	10	83	Excellent
Monzel et al., 2023 [79]	1	1	1	1	2	1	1	1	9	75	Good
Monzel et al., 2023 [93]	1	1	2	1	2	1	1	1	10	83	Excellent
Monzel et al., 2023 [30]	1	1	1	1	2	1	1	1	9	75	Good
Monzel et al., 2023 [96]	1	1	2	1	2	1	1	NA	9	82	Excellent
Monzel et al., 2022 [61]	1	1	1	1	2	1	1	1	9	75	Good
Monzel et al., 2021 [73]	1	1	2	1	2	1	1	1	10	83	Excellent
Palermo et al., 2022 [67]	1	1	1	1	2	1	1	0	8	67	Good
Pounder et al., 2024 [53]	1	1	1	1	2	1	1	1	9	75	Good
Pounder et al., 2022 [58]	1	1	2	1	2	1	1	1	10	83	Excellent
Purkart et al., 2024 [123]	1	1	2	1	0	1	1	1	8	67	Good

Reeder, 2022 [27]	1	0	1	1	2	1	1	1	8	67	Good
Siena & Simons, 2024 [63]	1	1	2	1	1	1	1	1	9	75	Good
Smyth et al., 2019 [37]	1	1	1	1	2	1	1	1	9	75	Good
Speed et al., 2024 [77]	1	1	1	1	1	1	1	1	8	67	Good
Takahashi & Gyoba, 2021 [49]	0	1	1	1	1	1	1	NA	6	55	Fair
Takahashi et al., 2023 [31]	1	1	1	1	2	1	1	1	9	75	Good
Thorudottir et al., 2020 [41]	1	1	2	1	2	1	1	0	9	75	Good
Toftness, 2022 [122]	1	1	1	1	1	1	1	1	8	67	Good
Wicken et al., 2021 [78]	1	2	2	1	2	1	1	1	11	92	Excel-lent
Wittmann & Satirer, 2022 [52]	1	1	2	1	2	1	1	1	10	83	Excel-lent
Zeman et al., 2020 [4]	1	1	0	1	2	1	1	1	8	67	Good
Zeman et al., 2015 [5]	1	1	0	1	2	1	1	1	8	67	Good
Zhao et al., 2020 [70]	1	2	2	1	2	1	1	1	11	92	Excel-lent

Note: NA, Not Applicable. The overall score for each research was calculated by summing the scores of the eight dimensions assessed and expressing them as a percentage of the maximum score (for some studies, part of the dimensions did not apply to be assessed (NA), therefore the maximum score may differ between studies). Based on the score in percentage, the following quality scores were excellent (score⩾81%), good (61-80%), fair (41-60%), poor (21-40%), or very poor (⩽20%).

Table S3. Prevalence.

Study	Sample (Mean age)	Assessing tools (Criteria of Aphantasia)	Key findings
Gouveia, 2023 [32]	Undergraduate students: 1627 (20.76)	VVIQ (16-32)	The prevalence of aphantasia was 5.9%.
Beran et al., 2023 [33]	Adults: 5010 (NA)	VVIQ (Lower than 24)	The prevalence of aphantasia was 1.5%.
Dance et al., 2022 [29]	Undergraduate students: 502 (19.93)<br>General population: 502 (36.55)	VVIQ (16-32)	The prevalence of aphantasia was 3.9%.<br>The prevalence of absent imagery was 0.8%.
Gulyas et al., 2022 [34]	Age12-16 Hungarian: 80 (14.39)<br>Age 21-30 Hungarian: 518 (26.91)<br>Age 31-40 Hungarian: 787 (35.49)<br>Age 41-50 Hungarian: 648 (44.90)<br>Age 51-60 Hungarian: 219 (54.26)	VVIQ (Lower than 20)	Vividness of visual imagery declined with the growth of age, especially in males.
Smyth et al., 2019 [37]	University students: 223 (NA)	VVIQ (16)	The prevalence was 0.9%. No significant differences in the VVIQ scores between different schools. Vividness of visual imagery had a positive relationship with age.

Table S4. Performance on computer tasks.

Study	Sample (Mean age)	Assessing tools	Key findings
Keogh & Pearson, 2024 [23]	Aphantasics: 55 (NA)	Binocular rivalry imagery paradigm	Aphantasics showed very limited image-based binocular rivalry priming.
Kay et al., 2022 [25]	Psychology students: 42<br>Aphantasics: 19 (35.8)	Pupillary response task<br>Binocular rivalry task	Aphantasics did not show imagery pupillary light response.
Reeder, 2022 [27]	1810 (23.993 across 1597 participants)	Ganzflicker experience	Individuals who completely lack visual imagery were much unlikely to experience pseudo-hallucination during the Ganzflicker stimulation than individuals who have low imagery vividness.
Konigsmark et al., 2021 [26]	Internet volunteers: 178 (27.180)<br>Psychology students: 28 (21.607)<br>(Contain 143 aphantasics and 63 typical imagers)	Ganzflicker experience	Aphantasics were less likely to experience complex and vivid pseudo-hallucinations.
Purkart et al., n.d. [123]	Aphantasics: 89 (34.7)<br>Control group: 62 (33.2)	Implicit priming task<br>Explicit priming task	Aphantasics did not show any priming effects in both tasks.
Keogh& Pearson, 2018 [13]	Aphantasics: 15 (NM)<br>Control group: 209 (NM)	Binocular rivalry imagery paradigm	Aphantasics showed very limited image-based binocular rivalry priming.

Table S5. Acquired aphantasia.

Study	Sample (Mean age)	Key findings
Bumgardner et al., 2021 [44]	Aphantasic: 1 (62)	Individual with refractory IgG kappa multiple myeloma acquired aphantasia after receiving an autologous stem cell transplant following high-dose melphalan.
Gaber & Eltemamy, 2021 [45]	Aphantasic: 1 (59)	Individual acquired aphantasic after contracting mild COVID-19.
Knowles et al., 2021 [42]	Aphantasics: 88 (NA)	Both neurological and psychological disorder, such as head injury, affective disorder, and stroke, could cause acquired aphantasia.

Table S6. Non-visual imagery ability.

Study	Sample (Mean age)	Assessing tools	Type	Key findings
Pounder et al., 2024 [53]	Aphantasics:29 (38y1m)<br>Control group: 30 (39y1m)	Bucknell Auditory Imagery Scale (BAIS)<br>Goldsmiths Musical Sophistication Index (Gold-MSI)<br>Musical pitch imagery task<br>Voice task	Auditory imagery	Controls with typical imagery scored significantly higher than participants with aphantasia in BAIS.<br>No difference in the performance on auditory imagery task was found between aphantasics and control groups.
Takahashi et al.,2023 [31]	Individuals: 2871 (38.3)	The Questionnaire upon Mental Imagery (QMI)	All modalities of imagery	Some participants reported less imagery in other imagery, whereas others only lacked visual imagery.
Dawes et al., 2024 [54]	Aphantasia sample1: 964 (39.24)<br>Aphantasia sample2: 1148 (40.00)	The Questionnaire upon Mental Imagery	All modalities of imagery	Aphantasia is a heterogenous phenomenon. There are two subtypes of aphantasia: visual aphantasia and multisensory aphantasia
Wittmann & Satirer, 2022 [52]	Aphantasics: 55 (37.6)<br>Control group: 41 (33.4)	Auditory Imagery;<br>Questionnaire	Auditory imagery	Lower auditory imagery was also found the aphantasic group.
Hinwar & Lambert, 2021 [51]	Adults: 128 (NM)	Bucknell Auditory Imagery Scale-Vividness (BAIS-V);<br>Bucknell Auditory Imagery Scale—Control (BAIS-C)	Auditory imagery	The majority of people who self-reported aphantasics also reported having little to no auditory imagery.
Dance Ward, & Simner, 2021 [48]	Aphantasics: 164 (42.35)<br>Control group: 138 (37.39);<br>Undergraduate students: 83 (19.87);<br>Aphantasics: 56 (33.66)<br>Control group: 56 (29.84)	Plymouth Sensory Imagery Questionnaire (Psi-Q);<br>Glasgow Sensory Questionnaire (GSQ);<br>Clarity of Auditory Imagery Scale (CAIS);<br>Vividness of Olfactory Imagery Questionnaire (VOIQ)；	All modalities of imagery	Aphantasics reported impaired imagery in a variety of categories.

		Adapted Shortened Betts' Questionnaire Upon Mental Imagery;<br>Vividness of Movement Imagery Questionnaire 2		
Dawes et al., 2020 [6]	Aphantasics: 267 (33.97)<br>Control group 1: 203 (33.82)<br>Control group 2: 197 (19.33)	The Questionnaire upon Mental Imagery (QMI)	All modalities of imagery	Aphantasic individuals reported less imagery in other imagery modalities.
Takahashi & Gyoba, 2021 [49]	Aphantasic: 1 (49)	The Questionnaire upon Mental Imagery (QMI);<br>Auditory Imagery Questionnaire (AIQ)	All modalities of imagery	Aphantasic individual reported less or deficit of imagery in other imagery modalities.
Zeman et al., 2020 [4]	Aphantasics: 2000 (41.31)<br>Hyperphantasics: 200 (41.87)<br>Control group:200 (56.80)	Self-designed question, the Imagery Questionnaire (IQ)	All modalities of imagery	According to 54.2% of aphantasic participants, all imagery modalities were weak.

Table S7. Memory.

Study	Sample (Mean age)	Assessing tools	Type	Key findings
Dando et al., 2023 [59]	Aphantasics: 60 (35.20)<br>Control group: 60 (32.02)	Interview	Episodic memory	Compared to controls, aphantasics recalled 30% less correct information, and their narratives were less complete.
Siena & Simons, 2024 [63]	Aphantasics: 20 (26.70)<br>Control group: 27 (27.73)	Custom 3D object and spatial memory task	Objective memory	Aphantasic participants were found to be unimpaired on all objective memory measures.
Beran et al., 2023 [33]	Adults: 5010 (NA)	Memory task	Memory	Aphantasics showed a poorer performance in the memory task.
Wittmann & Satirer, 2022 [52]	Aphantasics: 55 (37.60)<br>Control group: 41 (33.4)	Associative memory task	Memory confidence	All associative memory tests revealed that aphantasic participants had a lower incidence of high-confidence hits.
Dawes et al., 2022 [60]	Aphantasics: 30 (35.73)<br>Control group: 30 (35.77)	Episodic Memory Imagery Questionnaire (EMIQ); Adapted Autobiographical Interview	Episodic memory<br>Autobiographical memory	Aphantasia showed a decreased capacity for episodic autobiographical memory.
Knight & Milton, 2022 [57]	Aphantasics: 20 (26.35)<br>Control group: 22 (25.70)	Working Memory Task	Visual working memory	Aphantasia group were not significantly different from the control group.
Pounder et al., 2022 [58]	Aphantasics: 20 (40y0m)<br>Control group: 20 (39y6m)	Cambridge Neuropsychological Test Automated Battery (CANTAB)	Declarative memory<br>Visual working memory	Aphantasic individuals did not significantly exhibit deficits in either visual working memory or declarative memory.

Monzel, Vetterlein & Reuter, 2023 [30]	Aphantasics: 156 (35.23)<br>Interjacent group: 38 (29.87)<br>Control group: 131 (28.88)	Questionnaire for the Assessment of Everyday Memory Performance (FEAG)	Everyday memory<br>Autobiographical memory	Scores on every day and autobiographical memory were significantly lower for those with aphantasia than for controls.
Keogh et al., 2021 [56]	Aphantasics: 21; 15; 12 (30.85; 29.92; 34.00)<br>Control group: 68; 26; 13 (19.65; 20.23; 27.92)	Visual working memory capacity orientation task;<br>Number working memory capacity task;<br>Spatial visual working memory task;<br>Singular image retro-cue visual working memory task;<br>WAIS-IV Working Memory Index (WMI);<br>WMS-IV Visual Working Memory Index (VWM)	Visual working memory	Individuals with aphantasia did not have reduced visual working memory.
Bainbridge et al., 2021 [17]	Aphantasics: 61 (NM)<br>Control group: 52 (NM)	Drawing Recall Experiment	Object and spatial memory	Aphantasics showed deficits in object but not spatial memory
Milton et al., 2021 [16]	Aphantasics: 24 (33.70)<br>Hyperphantasics: 25 (34.60)<br>Midrange imagery vividness: 20 (35.36)	Logical Memory Test;<br>Rey–Osterrieth Complex Figure Test;<br>Rey–Osterrieth Complex Figure Test;<br>Autobiographical Interview	Anterograde memory;<br>Recognition memory Autographical memory	Aphantasia was associated with a significant decline in performance on demanding tests of autobiographical memory but not with a decline in anterograde memory or recognition memory.

Monzel et al., 2022 [61]	Aphantasics: 67 (30.94)<br>Control group: 32 (27.56)	Memory Task	Long-term memory<br>Short-term memory	Aphantasics had worse performance in visual and verbal long-term memory and short-term memory tests.
Dawes et al., 2020 [6]	Aphantasics: 267 (33.97)<br>Control group 1: 203 (33.82)<br>Control group 2: 197 (19.33)	The Episodic Memory Imagery Questionnaire (EMIQ);<br>The Survey of Autobiographical Memory (SAM);	Episodic memory;<br>Autographical memory;	Aphantasics was found to have significantly lower autobiographical and episodic memories.
Zeman et al., 2020 [4]	Aphantasics: 2000 (41.31)<br>Hyperphantasics: 200 (41.87)<br>Control group: 200 (56.80)	Self-designed question	Autobiographical memory	Autobiographical memory problem was more common in participants with aphantasia.
Ganczarek et al., 2020 [55]	Aphantasic: 1 (24)	Structured<br>specific interview;<br>Corsi Block-Tapping Task	Autobiographical memory;<br>Visuospatial working memory	Working memory performance was not affected, but autobiographical memory was found to be lacking.
Jacobs et al., 2018 [15]	Aphantasic: 1 (31y9m)<br>Control group: 11 (31y0m)	WAIS-IV Working Memory Index (WMI);<br>Working Memory Capacity (WMC) battery;<br>Visual Working Memory (WM)	Visual working memory	On most difficult visual working memory trials, aphantasic performed noticeably worse than controls.
Zeman et al., 2015 [5]	Aphantasics: 21 (NM)	Wechsler Memory Scale-IIIR	Autobiographical memory.	More over half of participants mentioned having trouble with autobiographical memory.

Table S8. Object and spatial imagery.

Study	Sample (Mean age)	Assessing tools	Key findings
Wittmann & Satirer, 2022 [52]	Aphantasics: 55 (37.6)<br>Control group: 41 (33.4)	Object and Spatial Imagery Questionnaire (OSIQ)	Aphantasic participants scored lower on the object subscale, but not on the spatial subscale.
Dawes et al., 2022 [60]	Aphantasics: 30 (35.73)<br>Control group: 30 (35.77)	Object and Spatial Imagery Questionnaire (OSIQ)	Aphantasic participants scored lower on the object subscale, but not on the spatial subscale.
Palermo et al., 2022 [67]	Adults: 490 (28.67)	Object and Spatial Imagery Questionnaire (OSIQ)	Object aphantasia and spatial aphantasia are the two subtypes of aphantasia.
Furman et al., 2022 [66]	Aphantasic: 1 (35)	VOSI	The participant did not have neither object nor spatial imagery.
Bainbridge et al., 2021 [17]	Aphantasics: 61 (NM)<br>Control group: 52 (NM)	Drawing Recall Experiement<br>Object and Spatial Imagery Questionnaire (OSIQ)	Aphantasic participants scored lower on the object scale, but not on the spatial scale.
Dawes et al., 2020 [6]	Aphantasics: 267 (33.97)<br>Control group 1: 203 (33.82)<br>Control group 2: 197 (19.33)	The Object and Spatial Imagery Questionnaire (OSIQ)	Aphantasic participants scored lower on the object scale, but not on the spatial scale.
Ganczarek et al., 2020 [55]	Aphantasic: 1 (24)	Object-Spatial Imagery and Verbal Questionnaire (OSIVQ)<br>Corsi Block-Tapping Task	Aphantasic had a poor object imagery score but a better spatial imagery score.
Keogh& Pearson, 2018 [13]	Aphantasics: 15 (NM)<br>Control group: 209 (NM)	Object and spatial imagery questionnaire (OSIQ)	Aphantasics had a poor object imagery score but a better spatial imagery score than controls.
Crowder, 2018 [65]	Aphantasics: 40 (43.20)<br>Non-aphantasia group: 79 (28.32)	Vividness of Object Spatial Imagery Questionnaire (VOSIQ)	Significantly less vivid object and spatial imagery was reported by the aphantasia group.

Table S9. Atemporal and future imagination.

Study	Sample (Mean age)	Assessing tools	Type	Key findings
Dawes et al., 2022 [60]	Aphantasics: 30 (35.73)<br>Control group: 30 (35.77)	Adapted Autobiographical Interview	Atemporal and future Imagination	Aphantasics produced considerably less episodic details than controls for both past and future events.
Milton et al., 2021 [16]	Aphantasics: 24 (33.70)<br>Hyperphantasics: 25 (34.60)<br>Midrange imagery vividness: 20 (35.36)	Future and atemporal imagination tasks	Atemporal and future Imagination	Aphantasia group scored significantly lower.

Table S10. Mental rotation task performance.

Study	Sample (Mean age)	Assessing tools	Key findings
Kay et al., 2024 [71]	Aphantasics: 95 (46.63)<br>Control group: 114 (46.5)<br>Aphantasics: 150 (44.68)<br>Control group: 164 (44.54)	Shepard-Metzler task<br>Manikin test	Aphantasics showed slower but more accurate performance in classic mental rotation tasks.<br>Aphantasics tended to employ self-embodied and analytic rotation strategies.
Zhao et al., 2022 [70]	Aphantasic: 1 (71)<br>Control group: 13 (70.8)	Mental rotation task (Canonical letter trials; mirror-reversed letter trials)	In the absence of voluntary object imagery, aphantasia exhibited spatial transformation capacity.
Pounder et al., 2022 [58]	Aphantasics: 20 (40y0m)<br>Control group: 20 (39y6m)	Mental rotation task	Participants with aphantasia were not significantly different from participants with common imagery. There was a clear group difference in response times in the group who reported a severe deficit in visual imagery.
Furman et al., 2022 [66]	Aphantasic: 1 (35)	Mental rotation test	Aphantasic showed high controllability of mental images in mental rotation tasks.
Milton et al., 2021 [16]	Aphantasics: 24 (33.70)<br>Hyperphantasics: 25 (34.60)<br>Midrange imagery vividness: 20 (35.36)	Manikins test	No overall difference between the two groups.
Ganczarek et al., 2020 [55]	Aphantasic: 1(24)	Mental rotation test	The participant showed higher than usual score in the mental rotation test.
Crowder, 2018 [65]	Aphantasics: 40 (43.20)<br>Non-aphantasia group: 79 (28.32)	The Vandenberg-Kuse Mental Rotation Test - Redrawn	Aphantasia group had slower performance in the mental rotation test.

Table S11. Visual search ability.

Study	Sample (Mean age)	Assessing tools	Key findings
Monzel & Reuter, 2024 [74]	Aphantasics: 104 (NM)<br>Control group: 104 (31.66)	Visual search task	Aphantasia were significantly slower than controls in the visual search task.
Monzel et al., 2021 [73]	Aphantasics and Non-aphantasics pair: 531 (27.64); 325 (28.74)	Moriya's Task;<br>Spontaneous use of visual imagery visual search task	Aphantasics were slower in image trials than non-aphantasics.

Table S12. Emotion.

Study	Sample (Mean age)	Assessing tools	Type	Key findings
Speed et al., 2024 [77]	Aphantasics: 47 (43.67)<br>Control group: 51 (38.64)	Transformative reading scale<br>Story World Absorption Scale<br>Open questions about the story	Empathy and emotional engagement with story	Aphantasics experienced reduced emotional engagement and less sympathy for characters in the story.
Hashim et al., 2024 [80]	Aphantasics: 51 (53.60)<br>Control group: 51 (50.00)	5-point Likert scale felt emotional intensity rating	Felt emotional response to the music	Aphantasics had a lower emotional intensity.
Monzel, Keidel &Reuter, 2023 [79]	Adults: 2232 (25.97)<br>Aphantasics: 112 (35.75)<br>Control group: 120 (30.08)	Interpersonal Reactivity Index (IRI);<br>Fantasy scale;<br>Perspective taking scale;<br>Empathic concern scale;<br>Personal distress scale<br>Pictorial Empathy Test (PET)	Empathy	When reading stories, aphantasic people exhibited significant less empathy, but there was no difference when viewing photos or videos.
Monzel, Vetterlein & Reuter, 2023 [30]	Aphantasics: 156 (35.23)<br>Control group: 131 (28.88)<br>Interjacent group: 38 (29.87)	Aphantasia Distress Questionnaire (ADQ);<br>Patient Health Questionnaire Depression Scale (PHQ-9);<br>Marburg Questionnaire on Habitual Well-being (FW-7)<br>State–Trait Anxiety Inventory (STAI-T);	Distress;<br>Depression;<br>Anxiety;<br>Well-being;<br>Control for response	The influence on daily living activities and personal anguish was too small, with 34.7% of participants with aphantasia reporting distress.
Wicken et al., 2021 [78]	Aphantasics: 22 (33); 16 (NM)<br>Control group: 24 (23); 15 (NM)	Imagery experiment;<br>Perception experiment	Fear	When reading scary stories, aphantasic people exhibited significant less physiological terror, but there was no difference when viewing spooky photos.
Milton et al., 2021 [16]	Aphantasics: 24 (33.70)<br>Hyperphantasics: 25 (34.60)<br>Midrange imagery vividness: 20 (35.36)	Hospital Anxiety and Depression Scale	Depression;<br>Anxiety	There was no overall significant difference between these groups.
Zeman et al., 2020 [4]	Aphantasics: 2000 (41.31)<br>Hyperphantasics: 200 (41.87)<br>Control group: 200 (56.80)	Self-designed question	Whether vividness of imagery was influenced by mood	Aphantasia group were less likely to report that mood influenced their imagery.

Table S13. Aphantasia and related disorders.

Study	Sample (Mean age)	Assessing tools	Type	Key findings
Takahashi et al.,2023 [31]	Individuals: 816 (41.8)	The 20-item Prosopagnosia Index (PI20)	Prosopagnosia	Aphantasia was significantly associated with lower face recognition ability.
Keogh et al., 2023 [81]	Aphantasics: 25 (35.56)<br>Control group: 23 (21.61)	Trauma Film;<br>PTSD Checklist for DSM-5 (PCL-5);<br>Peri Traumatic Emotion Ratings	PTSD	Aphantasic individuals experienced less intrusions immediately after watching trauma films.
Dance et al., 2023 [90]	Aphantasics: 52 (42.25)<br>Control group: 40 (41.23)<br>Imaging raters: 73 (35.15)<br>Aphantasics raters: 50 (42.94)	Face Composite Task;<br>Cambridge Face Memory Test;<br>Prosopagnosia Index;<br>The Autism Spectrum Quotient	Prosopagnosia<br>Autism	Aphantasics reported more traits associated with prosopagnosia.<br>Aphantasia individuals can still create facial composites from memory similar to control groups.<br>Aphantasia group had a significantly higher autism score.
Monzel Vetterlein, Hogeterp et al., 2023 [93]	Aphantasics: 65 (31,89)<br>Control group: 55 (29.05)	Cambridge Face Memory Test;<br>Cambridge Car Memory Test	Prosopagnosia	Aphantasics reported worse performance in face and objection recognition tasks than control group.<br>Visual imagery influenced visual recognition not only specifically in face recognition.
Palermo et al., 2022 [67]	Adults: 490 (28.67)	The 20-item prosopagnosia index (PI20)	Prosopagnosia	5.9% in the spatial aphantasia group had prosopagnosia
Furman et al., 2022 [66]	Aphantasic: 1 (35)	The 20-item Prosopagnosia Index (PI20)<br>The Exposure Based Face Memory Test (EBFMT)	Prosopagnosia	Score of aphantasia was not enough to be considered prosopagnosia
Milton et al., 2021 [16]	Aphantasics: 24 (33.70)<br>Hyperphantasics: 25 (34.60)<br>Midrange imagery vividness: 20	Autism Spectrum Quotient questionnaire;<br>Prosopagnosia Index (PI20);<br>Famous faces test	Autism;<br>Prosopagnosia	Compared to the controls, the aphantasia group had a significantly higher autism score. Face recognition difficulties was

	(35.36)			reported more commonly in aphantasia.
Dance Jaquiery, Eagleman et al., 2021 [87]	Aphantasics: 118 (38.47)<br>Control group: 118 (37.87)	Autism Quotient (AQ)	Autism	Aphantasics exhibited higher AQ scores and more frequently fall into the suggestive of autism spectrum.
Dawes et al., 2020 [6]	Aphantasics: 267 (33.97)<br>Control group 1: 203 (33.82)<br>Control group 2: 197 (19.33)	PTSD Checklist for DSM-5 (PCL-5)	Self-reported responses to stressful life events	In response to stressful life experiences, aphantasics did not seem to be immune to all types of trauma symptoms.
Zeman et al., 2020 [4]	Aphantasics: 2000 (41.31)<br>Hyperphantasics: 200 (41.87)<br>Control group: 200 (56.80)	Self-designed question	Prosopagnosia	Participants in the aphantasia group reported having substantially more trouble with face identification.
Thorudottir et al., 2020 [41]	Aphantasics: 1 (52)<br>Control group: 4 (62.5)	The WOF Test<br>Familiarity Decisions;<br>Naming of Familiar Item	Prosopagnosia	The aphantasic showed a deficit on most of individual face measures

Table S14. Neural Basis of Aphantasia.

Study	Sample (Mean age)	Assessing tools	Task	Key findings
Cabbai et al., 2024 [118]	Aphantasics: 24 (25.7)<br>Visualizer group: 26 (23.0)	fMRI	Passive listening task;<br>Voluntary imagery task	V1 representations could be distinguished from subjective imagery, and the precuneus played an important part in subjective imagery.
Dupont et al., 2024a [97]	Right-handed aphantasic: 14 (21)<br>Right-handed phantasic: 14 (23)	Single-pulse TMS	Motor imagery block;<br>Action observation	No increase in the amplitude of motor-evoked potentials in the aphantasia group
Dupont et al., 2024b [98]	Aphantasics: 16 (20)<br>Phantasics: 16 (23)	TMS	Action reading	Aphantasics had a deficit in motor system engagement during action reading.
Liu et al., 2023 [110]	Congenital aphantasia: 10 (28.69)<br>Typical imagers: 10 (29.28)	fMRI	Imagery and perceptual tasks	Imagery tasks activated the left frontal-parietal regions, the FIN, and areas in the ventral temporal cortex, which were similarly activated in the aphantasia and control groups. However, the connectivity between the FIN and frontoparietal regions was reduced in aphantasics.
Meng et al., 2023 [117]	Aphantasics: 14 (22.7)<br>Control group: 18 (23.9)	fMRI	Imagery generation task;<br>Passive viewing task	Compared to the typical controls, aphantasics' early visual areas in the two hemispheres showed different neural activation strengths and patterns.
Monzel, Leelaarporn, et al., 2023 [96]	Aphantasics: 14 (31.47)<br>Control group: 16 (28.19)	Resting State fMRI<br>Task-Based fMRI	Autobiographical memory retrieval task;<br>Simple math task	Aphantasic group exhibited decreased activation in the hippocampus and increased activation in the visual-perceptual cortex during an autobiographical memory task.
Furman et al., 2022 [66]	Aphantasic: 1 (35)	High-density (64 channel) EEG	Visual imagery task	Aphantasic showed the evoking phase from the left temporal area while lacking

				activation of the occipital and left anterior parietal areas when evoking mental images.
Milton et al., 2021 [16]	Aphantasics: 24 (33.70)<br>Hyperphantasics: 25 (34.60)<br>Midrange imagery vividness: 20 (35.36)	fMRI	Task including Perception–Control–Imagery blocks.	Individuals with hyperphantasia exhibited stronger connectivity between the prefrontal regions and visual-occipital network compared to the aphantasia group. When comparing visualization and perception of famous faces and places, individuals with hyperphantasia and the control group exhibited greater frontal and parietal activation compared to the aphantasia group.
Thorudottir et al., 2020 [41]	Aphantasics: 1 (52)<br>Control group: 4 (62.5)	Structural MRI	The WOF Test<br>Familiarity Decisions;<br>Naming of Familiar Item	Selective lesions in a specific area of the left fusiform gyrus and a portion of the right lingual gyrus were found in the aphantasic.
Fulford et al., 2018 [14]	High-vividness: 14 (NA)<br>Low-vividness: 15 (NA)	fMRI	Task including Perception–Imagery-Perception Control-Imagery Control blocks.	Individuals with more vivid mental images may stimulate the brain more selectively.<br>Posterior brain regions, including higher order visual association cortices, regions of posterior cingulate and precuneus and the MTL, were positively associated with vividness of visual imagery.

Table S15. Others.

Study	Sample (Mean age)	Assessing tools	Type	Key findings
Monzel et al., 2024 [120]	Aphantasics: 118 (36.19)<br>Control group: 119 (30.09)	Verbal overshadowing paradigm;<br>Adjusted strategy questionnaire	Verbal overshadowing	No verbal overshadowing effect was found in the aphantasia group.<br>Aphantasics may less use imagination-based strategies.
Balas, 2024 [165]	Aphantasics: 63 (NA)<br>Control group: 52 (NA)	Image Database	Mid-level characteristics of drawing	Aphantasics were more likely to create sparser drawings.<br>Visual imagery vividness influenced the presentation of a few mid-level characteristics.
Liu & Bartolomeo, 2023 [91]	Aphantasics: 44 (NA)<br>Typical imagers: 42 (NA)<br>Individuals with unusually vivid imagery ability: 31 (NA)	VVIQ;<br>Visual imagery tasks;<br>Visual perception tasks	Visual mental imagery<br>Visual perception	Aphantasics showed slower speed in visual imagery and perception tasks, while the accuracy was not be affected.
Toftness, 2022 [122]	Aphantasics: 95 (NA)<br>Control group:120 (NA)	Total Self-Reported Imagery Use (TSIU);<br>Backwards Spelling Task;<br>Snowy Pictures Task;<br>Tail-length Task;<br>Square Donut Scanning Task;<br>Change Identification Task	Performance on cognitive tasks	Aphantasics used different strategies to deal with cognitive task.
Keogh & Pearson, 2021 [19]	Aphantasics: 10 (27.8)<br>Control group: 10 (29.5)<br>Aphantasics: 15 (33.33)<br>Control group: 15 (20.67)	Imagery task;<br>Attention task;<br>Binocular rivalry task	Attentional templates	Aphantasics only showed feature-based attention, but not attentional templates.
Dance Ward, & Simner, 2021 [48]	Aphantasics: 164 (42.35)<br>Control group: 138 (37.39);	Glasgow Sensory Questionnaire (GSQ);<br>Pattern Glare Task	Sensory sensitivity	Aphantasics reported lower sensory sensitivity.

	Undergraduate students: 83 (19.87);<br>Aphantasics: 56 (33.66)<br>Control group: 56 (29.84)			
Fielding et al., 2020 [38]	Undergraduate macroeconomics student: 75 (20.3)	VVIQ<br>Macroeconomics exam	Economics academic performance	Imagery vividness was positively correlated with exam performance in male participants.
